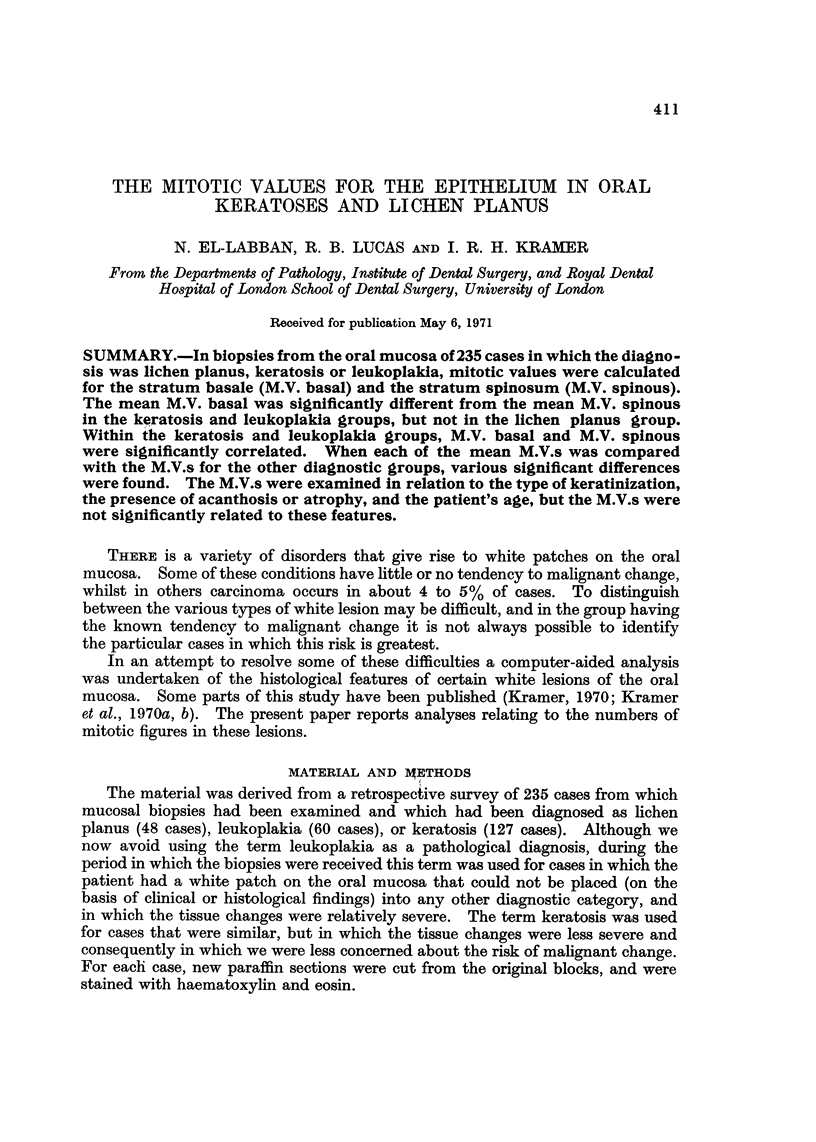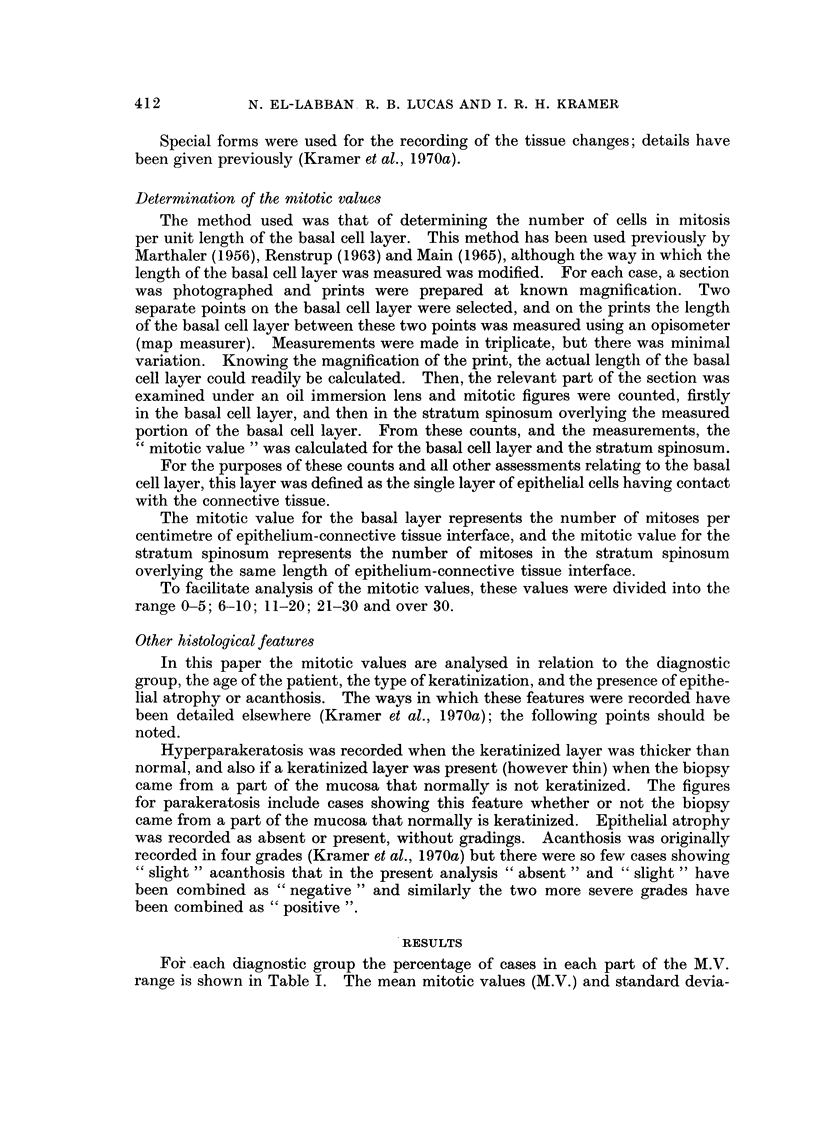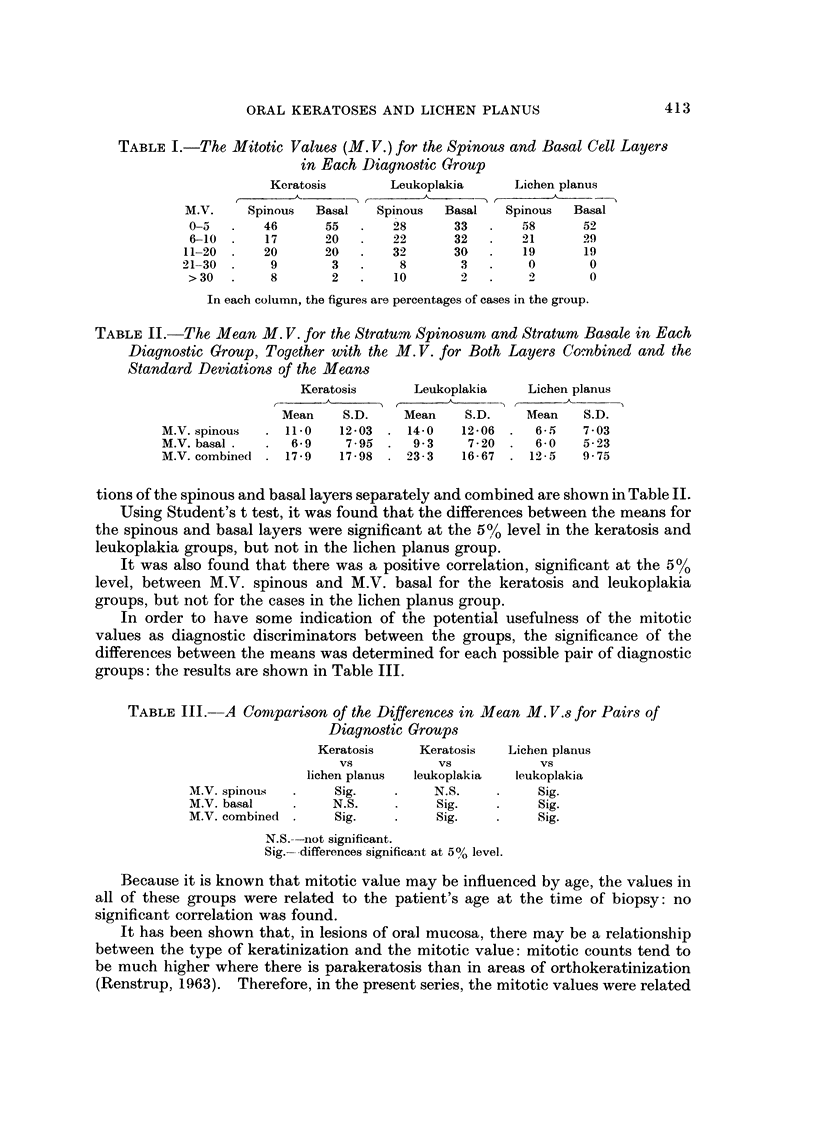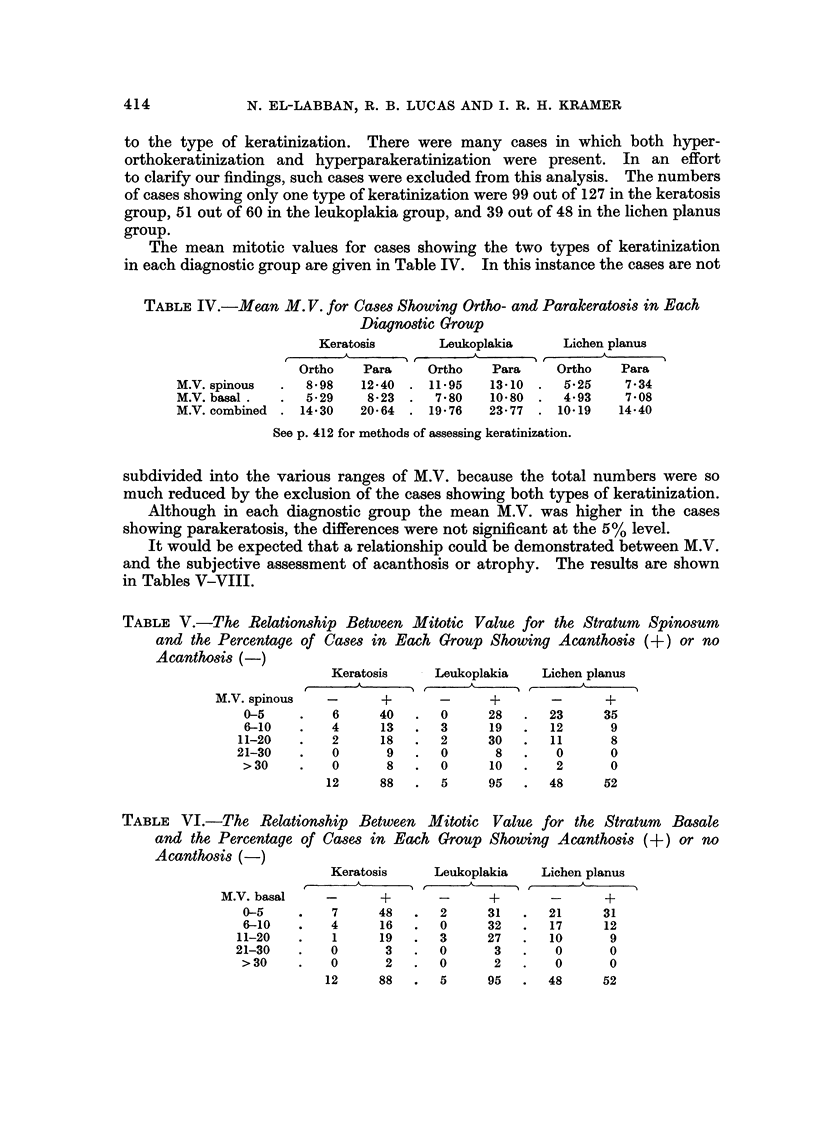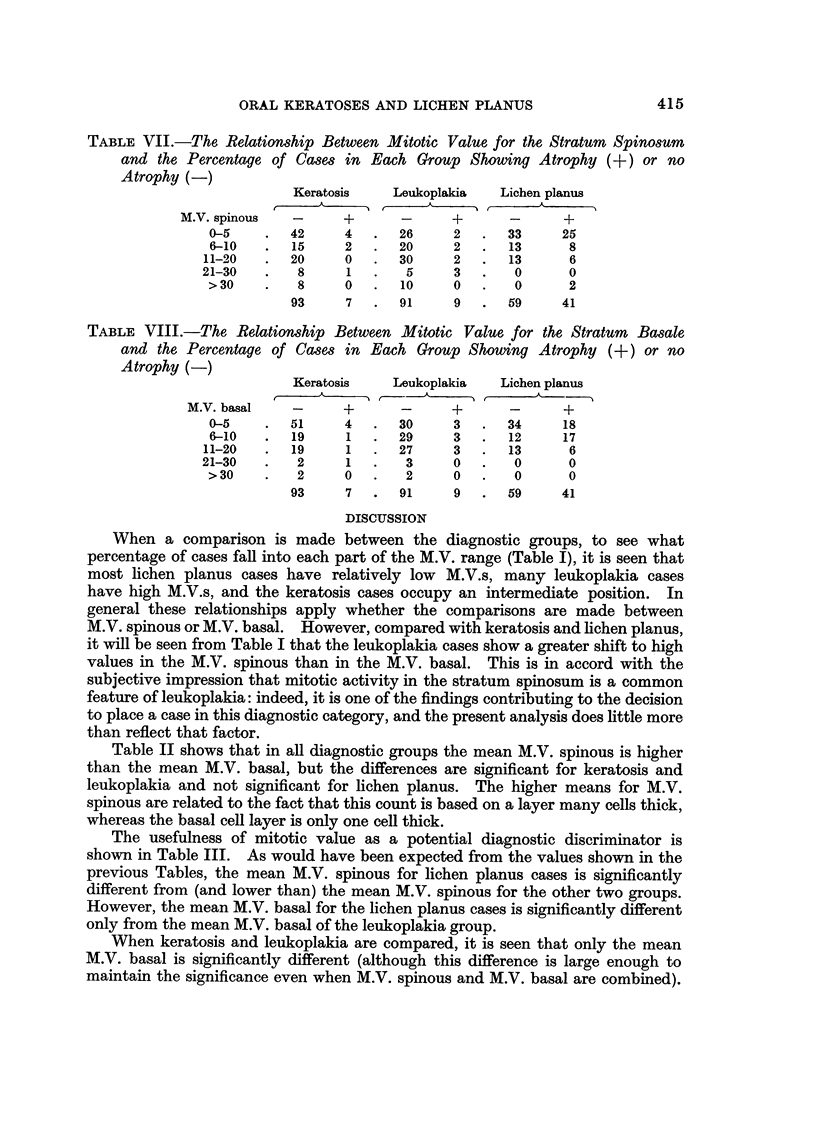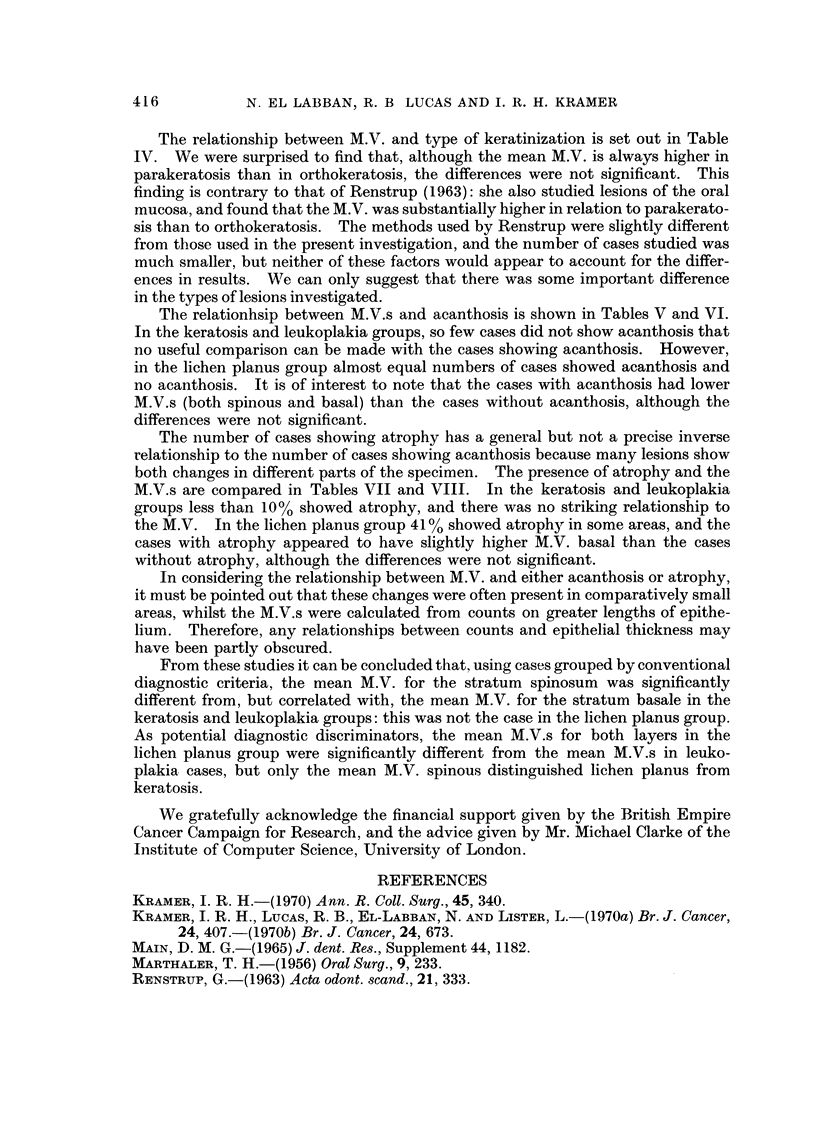# The Mitotic Values for the Epithelium in Oral Keratoses and Lichen Planus

**DOI:** 10.1038/bjc.1971.52

**Published:** 1971-09

**Authors:** N. El-Labban, R. B. Lucas, I. R. H. Kramer

## Abstract

In biopsies from the oral mucosa of 235 cases in which the diagnosis was lichen planus, keratosis or leukoplakia, mitotic values were calculated for the stratum basale (M.V. basal) and the stratum spinosum (M.V. spinous). The mean M.V. basal was significantly different from the mean M.V. spinous in the keratosis and leukoplakia groups, but not in the lichen planus group. Within the keratosis and leukoplakia groups, M.V. basal and M.V. spinous were significantly correlated. When each of the mean M.V.s was compared with the M.V.s for the other diagnostic groups, various significant differences were found. The M.V.s were examined in relation to the type of keratinization, the presence of acanthosis or atrophy, and the patient's age, but the M.V.s were not significantly related to these features.


### THE MITOTIC VALUES FOR THE EPITHELIUM IN ORAL KERATOSES AND LICHEN PLANUS

N. EL-LABBAN, R. B. LUCAS AND I. R. H. KRAMER

*From the Departments of Pathology, Institute of Dental Surgery, and Royal Dental Hospital of London School of Dental Surgery, University of London*

Received for publication May 6, 1971

**SUMMARY.—In biopsies from the oral mucosa of 235 cases in which the diagnosis was lichen planus, keratosis or leukoplakia, mitotic values were calculated for the stratum basale (M.V. basal) and the stratum spinosum (M.V. spinous). The mean M.V. basal was significantly different from the mean M.V. spinous in the keratosis and leukoplakia groups, but not in the lichen planus group. Within the keratosis and leukoplakia groups, M.V. basal and M.V. spinous were significantly correlated. When each of the mean M.V.s was compared with the M.V.s for the other diagnostic groups, various significant differences were found. The M.V.s were examined in relation to the type of keratinization, the presence of acanthosis or atrophy, and the patient's age, but the M.V.s were not significantly related to these features.**

THERE is a variety of disorders that give rise to white patches on the oral mucosa. Some of these conditions have little or no tendency to malignant change, whilst in others carcinoma occurs in about 4 to 5% of cases. To distinguish between the various types of white lesion may be difficult, and in the group having the known tendency to malignant change it is not always possible to identify the particular cases in which this risk is greatest.

In an attempt to resolve some of these difficulties a computer-aided analysis was undertaken of the histological features of certain white lesions of the oral mucosa. Some parts of this study have been published (Kramer, 1970; Kramer *et al.*, 1970*a*, *b*). The present paper reports analyses relating to the numbers of mitotic figures in these lesions.

### MATERIAL AND METHODS

The material was derived from a retrospective survey of 235 cases from which mucosal biopsies had been examined and which had been diagnosed as lichen planus (48 cases), leukoplakia (60 cases), or keratosis (127 cases). Although we now avoid using the term leukoplakia as a pathological diagnosis, during the period in which the biopsies were received this term was used for cases in which the patient had a white patch on the oral mucosa that could not be placed (on the basis of clinical or histological findings) into any other diagnostic category, and in which the tissue changes were relatively severe. The term keratosis was used for cases that were similar, but in which the tissue changes were less severe and consequently in which we were less concerned about the risk of malignant change. For each case, new paraffin sections were cut from the original blocks, and were stained with haematoxylin and eosin.

Special forms were used for the recording of the tissue changes; details have been given previously (Kramer *et al.*, 1970*a*).

*Determination of the mitotic values*

The method used was that of determining the number of cells in mitosis per unit length of the basal cell layer. This method has been used previously by Marthaler (1956), Renstrup (1963) and Main (1965), although the way in which the length of the basal cell layer was measured was modified. For each case, a section was photographed and prints were prepared at known magnification. Two separate points on the basal cell layer were selected, and on the prints the length of the basal cell layer between these two points was measured using an opisometer (map measurer). Measurements were made in triplicate, but there was minimal variation. Knowing the magnification of the print, the actual length of the basal cell layer could readily be calculated. Then, the relevant part of the section was examined under an oil immersion lens and mitotic figures were counted, firstly in the basal cell layer, and then in the stratum spinosum overlying the measured portion of the basal cell layer. From these counts, and the measurements, the " mitotic value " was calculated for the basal cell layer and the stratum spinosum.

For the purposes of these counts and all other assessments relating to the basal cell layer, this layer was defined as the single layer of epithelial cells having contact with the connective tissue.

The mitotic value for the basal layer represents the number of mitoses per centimetre of epithelium-connective tissue interface, and the mitotic value for the stratum spinosum represents the number of mitoses in the stratum spinosum overlying the same length of epithelium-connective tissue interface.

To facilitate analysis of the mitotic values, these values were divided into the range 0–5; 6–10; 11–20; 21–30 and over 30.

*Other histological features*

In this paper the mitotic values are analysed in relation to the diagnostic group, the age of the patient, the type of keratinization, and the presence of epithelial atrophy or acanthosis. The ways in which these features were recorded have been detailed elsewhere (Kramer *et al.*, 1970*a*); the following points should be noted.

Hyperparakeratosis was recorded when the keratinized layer was thicker than normal, and also if a keratinized layer was present (however thin) when the biopsy came from a part of the mucosa that normally is not keratinized. The figures for parakeratosis include cases showing this feature whether or not the biopsy came from a part of the mucosa that normally is keratinized. Epithelial atrophy was recorded as absent or present, without gradings. Acanthosis was originally recorded in four grades (Kramer *et al.*, 1970*a*) but there were so few cases showing " slight " acanthosis that in the present analysis " absent " and " slight " have been combined as " negative " and similarly the two more severe grades have been combined as " positive ".

### RESULTS

For each diagnostic group the percentage of cases in each part of the M.V. range is shown in Table I. The mean mitotic values (M.V.) and standard devia-

TABLE I.—*The Mitotic Values (M.V.) for the Spinous and Basal Cell Layers in Each Diagnostic Group*

| | Keratosis | | Leukoplakia | | Lichen planus | |
|---|---|---|---|---|---|---|
| M.V. | Spinous | Basal | Spinous | Basal | Spinous | Basal |
| 0–5 | 46 | 55 | 28 | 33 | 58 | 52 |
| 6–10 | 17 | 20 | 22 | 32 | 21 | 29 |
| 11–20 | 20 | 20 | 32 | 30 | 19 | 19 |
| 21–30 | 9 | 3 | 8 | 3 | 0 | 0 |
| >30 | 8 | 2 | 10 | 2 | 2 | 0 |

In each column, the figures are percentages of cases in the group.

TABLE II.—*The Mean M.V. for the Stratum Spinosum and Stratum Basale in Each Diagnostic Group, Together with the M.V. for Both Layers Combined and the Standard Deviations of the Means*

| | Keratosis | | Leukoplakia | | Lichen planus | |
|---|---|---|---|---|---|---|
| | Mean | S.D. | Mean | S.D. | Mean | S.D. |
| M.V. spinous | 11·0 | 12·03 | 14·0 | 12·06 | 6·5 | 7·03 |
| M.V. basal | 6·9 | 7·95 | 9·3 | 7·20 | 6·0 | 5·23 |
| M.V. combined | 17·9 | 17·98 | 23·3 | 16·67 | 12·5 | 9·75 |

tions of the spinous and basal layers separately and combined are shown in Table II.

Using Student's t test, it was found that the differences between the means for the spinous and basal layers were significant at the 5% level in the keratosis and leukoplakia groups, but not in the lichen planus group.

It was also found that there was a positive correlation, significant at the 5% level, between M.V. spinous and M.V. basal for the keratosis and leukoplakia groups, but not for the cases in the lichen planus group.

In order to have some indication of the potential usefulness of the mitotic values as diagnostic discriminators between the groups, the significance of the differences between the means was determined for each possible pair of diagnostic groups: the results are shown in Table III.

TABLE III.—*A Comparison of the Differences in Mean M.V.s for Pairs of Diagnostic Groups*

| | Keratosis vs lichen planus | Keratosis vs leukoplakia | Lichen planus vs leukoplakia |
|---|---|---|---|
| M.V. spinous | Sig. | N.S. | Sig. |
| M.V. basal | N.S. | Sig. | Sig. |
| M.V. combined | Sig. | Sig. | Sig. |

N.S.—not significant.
Sig.—differences significant at 5% level.

Because it is known that mitotic value may be influenced by age, the values in all of these groups were related to the patient's age at the time of biopsy: no significant correlation was found.

It has been shown that, in lesions of oral mucosa, there may be a relationship between the type of keratinization and the mitotic value: mitotic counts tend to be much higher where there is parakeratosis than in areas of orthokeratinization (Renstrup, 1963). Therefore, in the present series, the mitotic values were related to the type of keratinization. There were many cases in which both hyperorthokeratinization and hyperparakeratinization were present. In an effort to clarify our findings, such cases were excluded from this analysis. The numbers of cases showing only one type of keratinization were 99 out of 127 in the keratosis group, 51 out of 60 in the leukoplakia group, and 39 out of 48 in the lichen planus group.

The mean mitotic values for cases showing the two types of keratinization in each diagnostic group are given in Table IV. In this instance the cases are not

TABLE IV.—*Mean M.V. for Cases Showing Ortho- and Parakeratosis in Each Diagnostic Group*

| | Keratosis | | Leukoplakia | | Lichen planus | |
|---|---|---|---|---|---|---|
| | Ortho | Para | Ortho | Para | Ortho | Para |
| M.V. spinous | 8·98 | 12·40 | 11·95 | 13·10 | 5·25 | 7·34 |
| M.V. basal | 5·29 | 8·23 | 7·80 | 10·80 | 4·93 | 7·08 |
| M.V. combined | 14·30 | 20·64 | 19·76 | 23·77 | 10·19 | 14·40 |

See p. 412 for methods of assessing keratinization.

subdivided into the various ranges of M.V. because the total numbers were so much reduced by the exclusion of the cases showing both types of keratinization.

Although in each diagnostic group the mean M.V. was higher in the cases showing parakeratosis, the differences were not significant at the 5% level.

It would be expected that a relationship could be demonstrated between M.V. and the subjective assessment of acanthosis or atrophy. The results are shown in Tables V–VIII.

TABLE V.—*The Relationship Between Mitotic Value for the Stratum Spinosum and the Percentage of Cases in Each Group Showing Acanthosis* (+) *or no Acanthosis* (—)

| | Keratosis | | Leukoplakia | | Lichen planus | |
|---|---|---|---|---|---|---|
| M.V. spinous | — | + | — | + | — | + |
| 0–5 | 6 | 40 | 0 | 28 | 23 | 35 |
| 6–10 | 4 | 13 | 3 | 19 | 12 | 9 |
| 11–20 | 2 | 18 | 2 | 30 | 11 | 8 |
| 21–30 | 0 | 9 | 0 | 8 | 0 | 0 |
| >30 | 0 | 8 | 0 | 10 | 2 | 0 |
| | 12 | 88 | 5 | 95 | 48 | 52 |

TABLE VI.—*The Relationship Between Mitotic Value for the Stratum Basale and the Percentage of Cases in Each Group Showing Acanthosis* (+) *or no Acanthosis* (—)

| | Keratosis | | Leukoplakia | | Lichen planus | |
|---|---|---|---|---|---|---|
| M.V. basal | — | + | — | + | — | + |
| 0–5 | 7 | 48 | 2 | 31 | 21 | 31 |
| 6–10 | 4 | 16 | 0 | 32 | 17 | 12 |
| 11–20 | 1 | 19 | 3 | 27 | 10 | 9 |
| 21–30 | 0 | 3 | 0 | 3 | 0 | 0 |
| >30 | 0 | 2 | 0 | 2 | 0 | 0 |
| | 12 | 88 | 5 | 95 | 48 | 52 |

TABLE VII.—*The Relationship Between Mitotic Value for the Stratum Spinosum and the Percentage of Cases in Each Group Showing Atrophy* (+) *or no Atrophy* (—)

| | Keratosis | | Leukoplakia | | Lichen planus | |
|---|---|---|---|---|---|---|
| M.V. spinous | — | + | — | + | — | + |
| 0–5 | 42 | 4 | 26 | 2 | 33 | 25 |
| 6–10 | 15 | 2 | 20 | 2 | 13 | 8 |
| 11–20 | 20 | 0 | 30 | 2 | 13 | 6 |
| 21–30 | 8 | 1 | 5 | 3 | 0 | 0 |
| >30 | 8 | 0 | 10 | 0 | 0 | 2 |
| | 93 | 7 | 91 | 9 | 59 | 41 |

TABLE VIII.—*The Relationship Between Mitotic Value for the Stratum Basale and the Percentage of Cases in Each Group Showing Atrophy* (+) *or no Atrophy* (—)

| | Keratosis | | Leukoplakia | | Lichen planus | |
|---|---|---|---|---|---|---|
| M.V. basal | — | + | — | + | — | + |
| 0–5 | 51 | 4 | 30 | 3 | 34 | 18 |
| 6–10 | 19 | 1 | 29 | 3 | 12 | 17 |
| 11–20 | 19 | 1 | 27 | 3 | 13 | 6 |
| 21–30 | 2 | 1 | 3 | 0 | 0 | 0 |
| >30 | 2 | 0 | 2 | 0 | 0 | 0 |
| | 93 | 7 | 91 | 9 | 59 | 41 |

### DISCUSSION

When a comparison is made between the diagnostic groups, to see what percentage of cases fall into each part of the M.V. range (Table I), it is seen that most lichen planus cases have relatively low M.V.s, many leukoplakia cases have high M.V.s, and the keratosis cases occupy an intermediate position. In general these relationships apply whether the comparisons are made between M.V. spinous or M.V. basal. However, compared with keratosis and lichen planus, it will be seen from Table I that the leukoplakia cases show a greater shift to high values in the M.V. spinous than in the M.V. basal. This is in accord with the subjective impression that mitotic activity in the stratum spinosum is a common feature of leukoplakia: indeed, it is one of the findings contributing to the decision to place a case in this diagnostic category, and the present analysis does little more than reflect that factor.

Table II shows that in all diagnostic groups the mean M.V. spinous is higher than the mean M.V. basal, but the differences are significant for keratosis and leukoplakia and not significant for lichen planus. The higher means for M.V. spinous are related to the fact that this count is based on a layer many cells thick, whereas the basal cell layer is only one cell thick.

The usefulness of mitotic value as a potential diagnostic discriminator is shown in Table III. As would have been expected from the values shown in the previous Tables, the mean M.V. spinous for lichen planus cases is significantly different from (and lower than) the mean M.V. spinous for the other two groups. However, the mean M.V. basal for the lichen planus cases is significantly different only from the mean M.V. basal of the leukoplakia group.

When keratosis and leukoplakia are compared, it is seen that only the mean M.V. basal is significantly different (although this difference is large enough to maintain the significance even when M.V. spinous and M.V. basal are combined).

The relationship between M.V. and type of keratinization is set out in Table IV. We were surprised to find that, although the mean M.V. is always higher in parakeratosis than in orthokeratosis, the differences were not significant. This finding is contrary to that of Renstrup (1963): she also studied lesions of the oral mucosa, and found that the M.V. was substantially higher in relation to parakeratosis than to orthokeratosis. The methods used by Renstrup were slightly different from those used in the present investigation, and the number of cases studied was much smaller, but neither of these factors would appear to account for the differences in results. We can only suggest that there was some important difference in the types of lesions investigated.

The relationhsip between M.V.s and acanthosis is shown in Tables V and VI. In the keratosis and leukoplakia groups, so few cases did not show acanthosis that no useful comparison can be made with the cases showing acanthosis. However, in the lichen planus group almost equal numbers of cases showed acanthosis and no acanthosis. It is of interest to note that the cases with acanthosis had lower M.V.s (both spinous and basal) than the cases without acanthosis, although the differences were not significant.

The number of cases showing atrophy has a general but not a precise inverse relationship to the number of cases showing acanthosis because many lesions show both changes in different parts of the specimen. The presence of atrophy and the M.V.s are compared in Tables VII and VIII. In the keratosis and leukoplakia groups less than 10% showed atrophy, and there was no striking relationship to the M.V. In the lichen planus group 41% showed atrophy in some areas, and the cases with atrophy appeared to have slightly higher M.V. basal than the cases without atrophy, although the differences were not significant.

In considering the relationship between M.V. and either acanthosis or atrophy, it must be pointed out that these changes were often present in comparatively small areas, whilst the M.V.s were calculated from counts on greater lengths of epithelium. Therefore, any relationships between counts and epithelial thickness may have been partly obscured.

From these studies it can be concluded that, using cases grouped by conventional diagnostic criteria, the mean M.V. for the stratum spinosum was significantly different from, but correlated with, the mean M.V. for the stratum basale in the keratosis and leukoplakia groups: this was not the case in the lichen planus group. As potential diagnostic discriminators, the mean M.V.s for both layers in the lichen planus group were significantly different from the mean M.V.s in leukoplakia cases, but only the mean M.V. spinous distinguished lichen planus from keratosis.

We gratefully acknowledge the financial support given by the British Empire Cancer Campaign for Research, and the advice given by Mr. Michael Clarke of the Institute of Computer Science, University of London.

### REFERENCES

KRAMER, I. R. H.—(1970) *Ann. R. Coll. Surg.*, **45**, 340.

KRAMER, I. R. H., LUCAS, R. B., EL-LABBAN, N. AND LISTER, L.—(1970*a*) *Br. J. Cancer*, **24**, 407.—(1970*b*) *Br. J. Cancer*, **24**, 673.

MAIN, D. M. G.—(1965) *J. dent. Res.*, Supplement 44, 1182.

MARTHALER, T. H.—(1956) *Oral Surg.*, **9**, 233.

RENSTRUP, G.—(1963) *Acta odont. scand.*, **21**, 333.